# Topographic signatures and manipulations of Fe atoms, CO molecules and NaCl islands on superconducting Pb(111)

**DOI:** 10.3762/bjnano.13.1

**Published:** 2022-01-03

**Authors:** Carl Drechsel, Philipp D’Astolfo, Jung-Ching Liu, Thilo Glatzel, Rémy Pawlak, Ernst Meyer

**Affiliations:** 1 Department of Physics, Universität Basel, Klingelbergstrasse 82, 4056 Basel, Switzerland

**Keywords:** carbon monoxide (CO), lateral manipulation, NaCl, scanning tunneling microscopy, superconductivity

## Abstract

Topological superconductivity emerging in one- or two-dimensional hybrid materials is predicted as a key ingredient for quantum computing. However, not only the design of complex heterostructures is primordial for future applications but also the characterization of their electronic and structural properties at the atomic scale using the most advanced scanning probe microscopy techniques with functionalized tips. We report on the topographic signatures observed by scanning tunneling microscopy (STM) of carbon monoxide (CO) molecules, iron (Fe) atoms and sodium chloride (NaCl) islands deposited on superconducting Pb(111). For the CO adsorption a comparison with the Pb(110) substrate is demonstrated. We show a general propensity of these adsorbates to diffuse at low temperature under gentle scanning conditions. Our findings provide new insights into high-resolution probe microscopy imaging with terminated tips, decoupling atoms and molecules by NaCl islands or tip-induced lateral manipulation of iron atoms on top of the prototypical Pb(111) superconducting surface.

## Introduction

The most exciting manifestation of topological superconductivity [1–3] is the Majorana zero mode (MZM), which has attracted a tremendous interest due to its non-Abelian quantum exchange statistics proposed as a key ingredient for topological quantum computing [4–6]. Topological superconductivity can intrinsically arise in the bulk of certain materials [7] or can be engineered at the interface between two materials, exhibiting particle–hole symmetry and spin–orbit interaction [8]. Among the most promising platforms to realize MZMs are semiconducting nanowires with large spin–orbit coupling [9–12] or atomic chains [13–18] in proximity to an *s*-wave superconductor. The realization of MZMs in two dimensions has been also observed in vortex cores on a proximitized topological insulator surface [19–20], in iron-based superconductors [7,21–22] or hybrid van der Waals heterostructures [23]. The fingerprint for MZMs in conductance measurements through the nanowire or in scanning tunneling spectroscopy (STS) is a zero-bias conductance peak occurring at boundaries and defects. Unfortunately, other structural peculiarities can also mimic such zero-bias anomalies, which eventually leads to severe misinterpretations. Therefore, the latest advances in scanning tunneling microscopy (STM) and atomic force microscopy (AFM) are required to accurately disentangle structural and electronic properties of atomic or molecular structures on these superconducting platforms.

STM/AFM generally allows for a controlled repositioning of adsorbates, both by lateral and vertical manipulations [24–26]. Atoms and molecules can be pushed or pulled laterally across a surface [25,27–28], but can also be picked up and dropped with the probing tip [29–30]. This offers the opportunity to design atomic structures with novel electronic properties [25,31–32]. Vertical manipulations enable the development of functionalized tips, obtained by picking up a single molecule from a surface. This has been an important milestone for low-temperature STM/AFM techniques since the CO tip nowadays enables systematic high-resolution measurements of surfaces, molecules and atoms [33–35].

It is, however, astonishing that most recent advances in manipulation experiments or contrast enhancement with functionalized tips are hitherto at their infancy, when studying a superconducting surface by STM/AFM. Although the earliest proposal for observing MZMs suggested to reposition Fe adatoms one by one with an STM tip in an one-dimensional fashion on an *s*-wave superconductor [10], this strategy has been primarily postponed in favor of self-assembly processes on Pb(110) surfaces [13–15,36]. Only recently, the successful manipulation of tens of Fe atoms has been reported on superconducting Re(0001) [16] and Ta(100)-O surfaces [37]. Despite being well established on many noble metals, the use of CO-terminated tips also remains quite scarce in the literature [38], which severely limits the use of AFM as imaging tool on superconductors.

Recently, Heinrich et al. have demonstrated the possibility to tune the magnetic anisotropy of a single porphyrin molecule by perturbing its ligand field with the STM probe [39–40]. These results not only suggest the importance of future manipulations experiments, but also shed new lights into the potential of decoupling atoms and molecules electronically from the underlying superconductors. With this prospect, we emphasize that, in addition to tip manipulations, the use of alkali halide islands, adsorbed on a superconducting surface and acting as a buffer layer, is another interesting field for research on topological superconductors [41–44].

In this work, we report on the topographic features of adsorbed CO molecules, NaCl layers and Fe adatoms on a superconducting Pb(111) surface, investigated with STM at 4.8 K. We show that CO molecules on Pb(111) are hardly visible in STM images due to their high diffusion induced by the tip even at low temperature. This differs distinctly from the adsorption on Pb(110), which has also been performed. In contrast, NaCl islands and single Fe atoms are more stable. Nevertheless, a general propensity for a tip-induced displacement of these adsorbates on the Pb(111) surface can be fulfilled. We believe that our results help to identify these adsorbates and constitute an important step for future experiments to perform high-resolution STM/AFM imaging with CO-terminated tips or in the electronic decoupling of atoms and molecules from the prototypical Pb(111) superconducting surface.

## Experimental

### Sample preparation

The Pb(111) single crystal, purchased from Mateck GmbH, was cleaned by several sputtering and annealing cycles in ultra-high vacuum (UHV). CO dosing on the cold substrate was done in the microscope chamber by increasing the pressure via a leak valve up to *p* ≈ 1 × 10^−7^ mbar for one minute. This leads to a surface coverage of about 0.1–0.3 monolayers, as we readily observed on noble metals such as Cu, Ag or Au [45–46]. Iron adatoms were evaporated in the microscope head on the substrate at a temperature below 15 K. NaCl was evaporated from a quartz crucible on samples kept at room temperature in the preparation chamber.

### Low-temperature scanning tunneling microscope

The experiments were performed using a low-temperature STM/AFM microscope (*T* = 4.8 K) from Omicron GmbH in UHV (*p* ≈ 1 × 10^−10^ mbar) operated with Nanonis RC5 electronics. The sensor is a tuning fork sensor in a qPlus design [47] operated in the frequency-modulation mode (resonance frequency *f*_0_ ≈ 25 kHz, spring constant *k* ≈ 1800 N/m, quality factor *Q* ≈ 14000, and oscillation amplitude *A* ≈ 0.5 Å). The tip mounted to the qPlus sensor consists of a 25 μm-thick PtIr wire, shortened and sharpened with a focused ion beam. A clean and sharp Pb tip was then prepared at low temperature by repeated indentations into the surface. STM images were acquired in constant-current mode with the bias voltage applied to the tip. All experimental data were analysed by using Gwyddion [48].

## Results and Discussion

### CO adsorption on Pb(111) and Pb(110)

**Figure 1:**
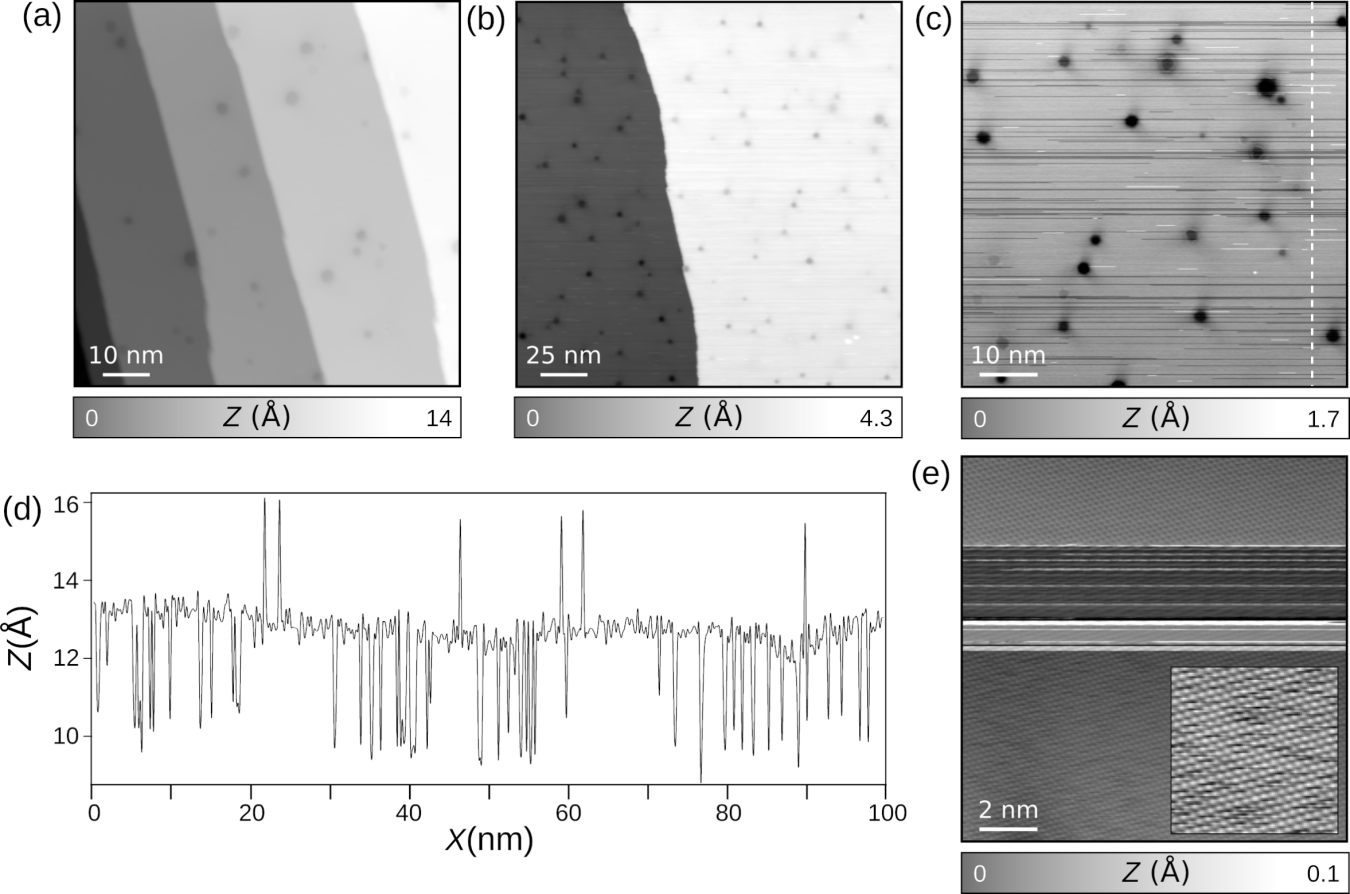
Carbon monoxide (CO) molecules adsorbed on Pb(111). (a) STM overview image of pristine Pb(111) (*V_t_* = −0.1 V, *I_t_* = 1 pA). (b) STM image after CO deposition. The estimated coverage is below 0.2 monolayers. (c) Close-up STM topography of CO molecules diffusing on the surface during scanning (*V_t_* = −0.1 V, *I_t_* = 40 pA). (d) Profile taken along the dashed white line of panel (c) showing spontaneous CO displacement under tip action. (e) Enhanced STM resolution (inset: more detailed image of the Pb(111) surface) resulting from the termination of the tip by a CO molecule (*V_t_* = −0.2 V, *I_t_* = 1 pA).

Figure 1 shows STM images of CO molecules adsorbed on Pb(111). With a lattice parameter of *a*_Pb_ = 4.95 Å, the height of monoatomic steps of the Pb(111) surface is expected to be *h_Pb_* = 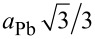 = 2.85 Å. Experimentally, a pristine Pb(111) sample (Figure 1a) shows, after sputtering and annealing cycles, typically steps of ≈2.7 Å, which thus corresponds to monoatomic steps. This is in contrast to Pb islands grown on surfaces that exhibit a double layer growth due to quantum size effect [49–51]. On the terraces, hexagonal dark spots are visible by STM, whose diameters vary between 1.5 and 5 nm with an apparent depression of 0.14 Å. They result from the interference of bulk electrons with trapped subsurface Ar gas bubbles after sputtering [52–53].

After CO dosing in the microscope chamber (see section “Sample preparation”), a coverage of 0.1–0.2 monolayers is expected to adsorb on the metal surface, as observed on different noble metals [45–46]. Figure 1b and Figure 1c show STM topographic images after such process. While the surface topography remains unchanged in comparison to Figure 1a, numerous scan instabilities are now present, which we attribute to CO molecules diffusing under gentle scan conditions (tunneling resistance of 200 GΩ). The STM profile (Figure 1d) taken along the white dashed line of Figure 1c shows several stochastic jumps, which we interpret as tip-induced displacements of single CO molecules [54–56]. We emphasize that the change of various scan parameters as well as tip indentations into the clean Pb surface were conducted to avoid such instabilities without noticeable improvements. Nevertheless, an unintentional CO tip termination could be achieved as shown by the enhancement of the STM resolution in Figure 1e. In comparison to vertical manipulations of CO on noble metals, we emphasize that CO-terminated tips on Pb(111) are much less stable, which severely limits the use of CO-terminated STM/AFM imaging on Pb(111). It should be noted that other tip terminations are also possible (such as with Xe), which we plan to explore in future work.

**Figure 2:**
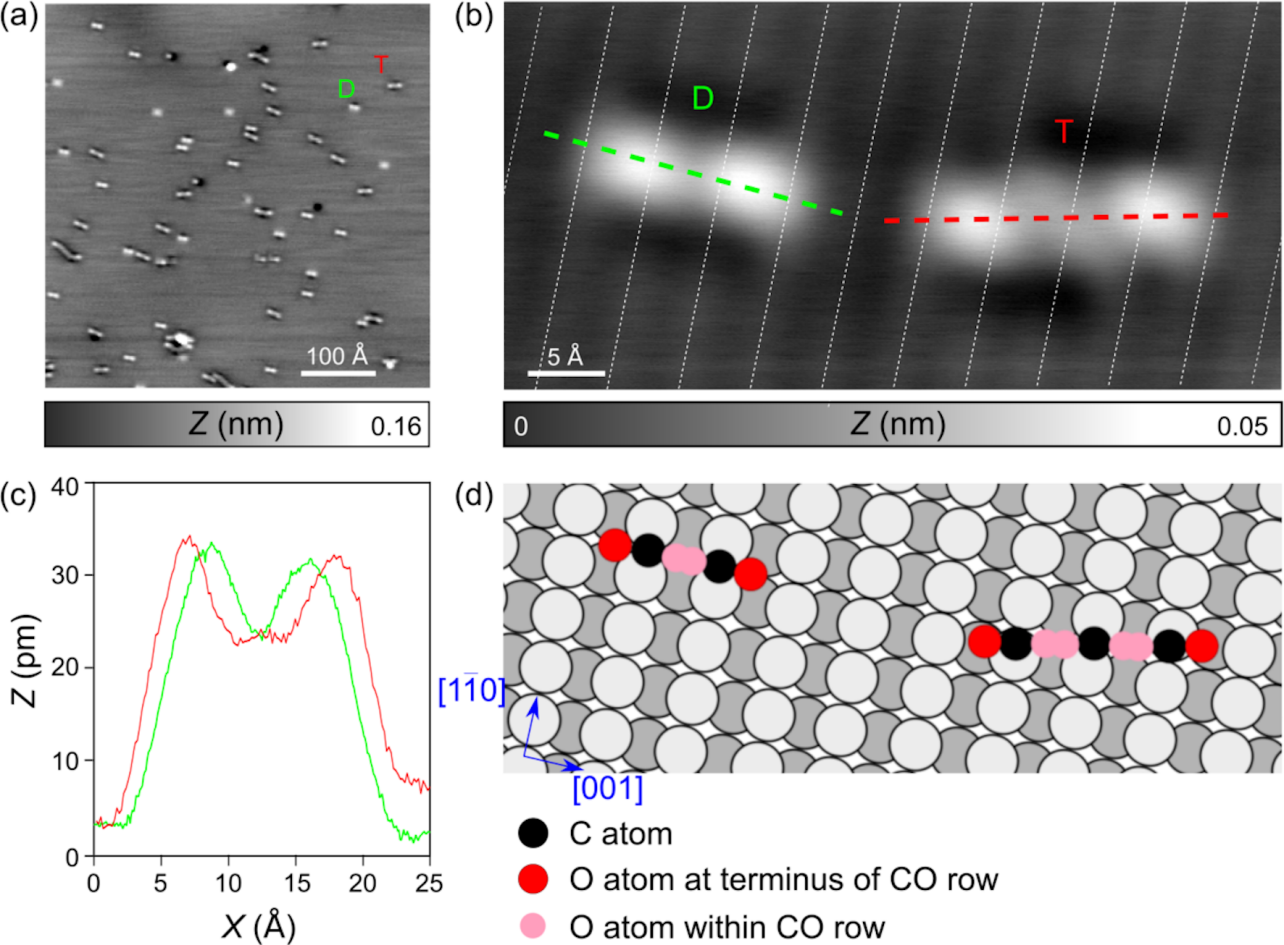
Adsorption of carbon monoxide (CO) molecules on Pb(110). (a) STM overview image of Pb(110) after adsorption of CO molecules (D: dimer, T: trimer, *V_t_* = −0.5 V, *I_t_* = 0.5 pA). (b) Closed-up STM image of a CO dimer and trimer (*V_t_* = −0.1 V, *I_t_* = 1 pA). (c) Profiles taken along the dashed lines of panel (b). (d) Sphere model of CO adsorbed on Pb(110). The CO molecules are standing up with protruding and tilted oxygen atoms (red, when tilted to the chain terminus; pink, when tilted to the center of the CO chain), white and dark gray spheres refers to the topmost and downmost Pb atoms of the Pb(110) reconstruction.

Similar CO adsorption experiments were also conducted on Pb(110) (Figure 2a). There, most CO molecules appear in STM images as linear aggregates of different lengths, aligned nearly perpendicular to the 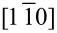 row direction of Pb(110). The dimer-like protrusions ((*D*) in Figure 2b) exhibit a length of ≈7 Å between maxima (Figure 2c), corresponding to the distance of *a*_Pb_ = 4.95 Å between two Pb(110) rows (dashed lines in Figure 2b). In agreement with [57], the additional length of ≈2 Å might be related to the tilting of the adsorbed CO molecules under the scanning tip as well as the tip convolution during imaging. The trimeric protrusion (*T*) is rotated by about 16° compared to the 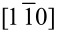 rows. Its length of ≈11 Å corresponds to about three Pb(110) atomic rows, the additional length of ≈1 Å is again imputed to tilted CO during tip scanning. Last, these protrusions have a slight apparent depression around them, which might be related to a strong interaction with the Pb lattice. While the D features are all aligned in the same direction, the orientation of the T features differs slightly. Both features have an apparent height of ≈0.3 Å, as extracted from the profile of Figure 2b, displayed in Figure 2c.

Overall, the CO adsorption on Pb(110) shows strong similarities with CO adsorbed on Cu(110) [58] and Cu(110)-(2 × 1)O [57,59]. In these works, CO assembled for low coverage and low temperature as monomers, dimers and occasionally trimers in agreement with our data, while longer chain configurations were observed at much higher temperatures on both surfaces. Dimer and chain structures were always aligned perpendicularly to the close-packed rows suggesting an attractive interaction along the [001] direction of the substrate [58]. CO adsorption sites were found with the C atom on top of Cu or Cu-O rows. Importantly, STM images on both substrates revealed local maxima between individual CO molecules constituting the chains like on Pb(110). These observations were suggested to be related to a charge density perturbation resulting from substrate-mediated attractive interaction between CO molecules. Note also that two tilted CO configurations of ±45° are supposed to coexist on the surface and convert rapidly upon scanning [57]. In their work supported by DFT, Feng et al. [57] further described the formation of CO rows by dipole–dipole interactions that can be repulsive for vertically adsorbed CO molecules [60–61] but are attractive in their tilted configurations [57]. We think that a very similar mechanism might govern the CO adsorption of the Pb(110) surface, leading to the observed chain structures with very similar contrast. If we transfer this model to our measurements, the adsorption of the CO molecules might take place on top of the 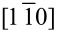 rows of the Pb(110) surface, as shown by the model in Figure 2d. For the dimer (D), the C atom is probably bonded to the Pb at the bridge sites of 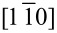 rows. The CO molecule is tilted similarly as on the Cu(110)-(2 × 1)O surface [57,59] and appears in STM above the trenches of the Pb(110) surface. For the trimer (T), the mutual interaction of the interior CO molecules might cause a slight mismatch with the Pb(110) layer, which explains the small deviation from the perpendicular alignment of the dimers. For longer CO aggregates, this deviation becomes even more apparent (see Figure 2a).

### Growth of NaCl islands on Pb(111)

**Figure 3:**
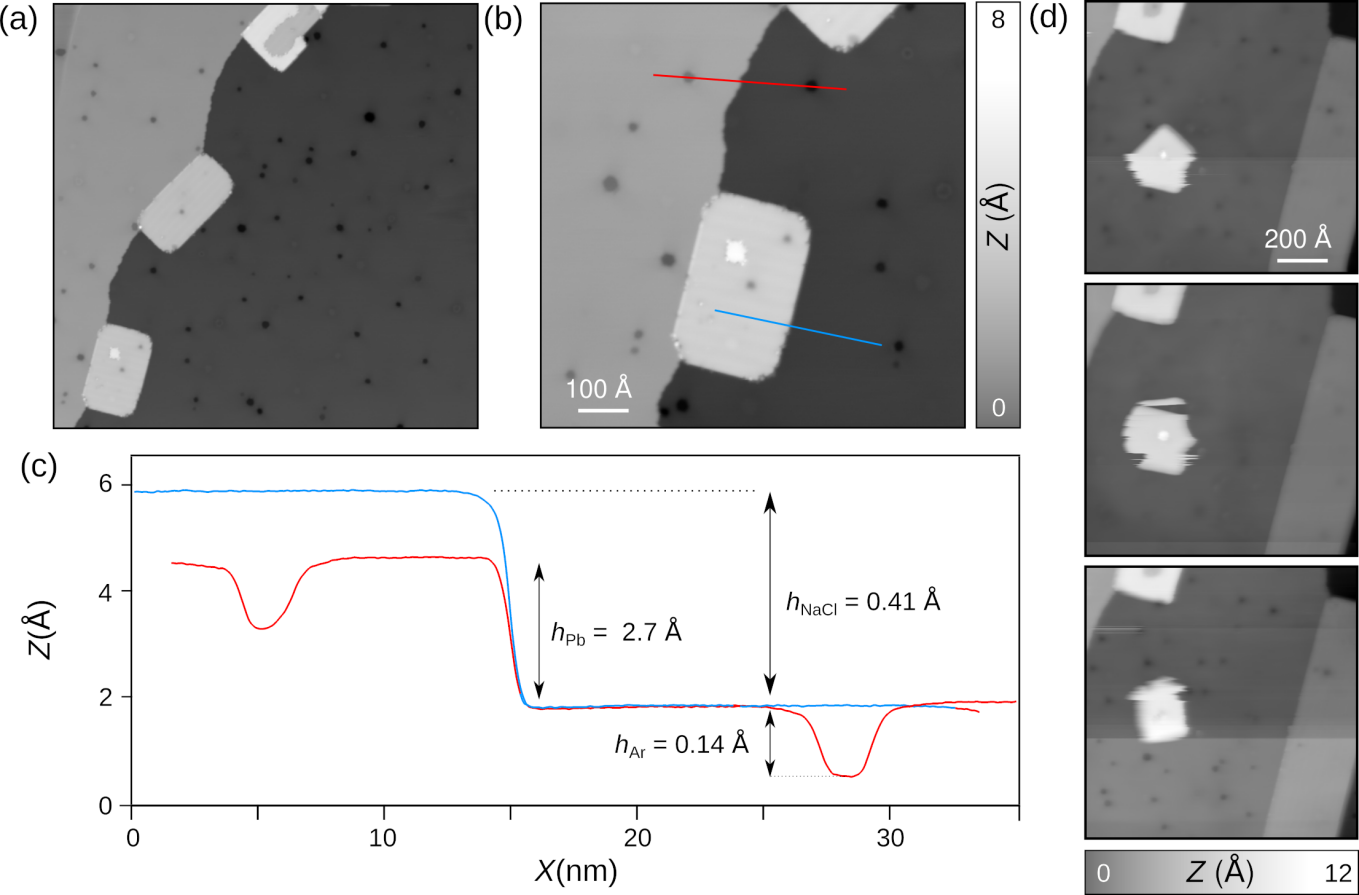
Adsorption of NaCl on Pb(111). (a, b) STM overview image of Pb(111) with quadratic NaCl islands adsorbed at step edges (*V_t_* = −0.4 V, *I_t_* = 1 pA). (c) Height profile extracted along the red and blue lines of panel (b). (d) Series of STM image showing the tip-induced rotation of an NaCl island (*V_t_* = −0.4 V, *I_t_* = 40 pA).

We next investigated the adsorption of NaCl on Pb(111) (Figure 3). Upon sublimation from a quartz crucible on a Pb(111) surface, which is kept at room temperature, NaCl forms, without any post-annealing, rectangular islands with round corners attached to Pb step edges (Figure 3a and Figure 3b). According to the profile, shown in Figure 3c, which was extracted along the red and blue lines of Figure 1b, the step heights are equal to *h*_NaCl_ = 4.1 Å. This corresponds to a NaCl bilayer and is in agreement with the reported growth of NaCl islands on Cu(111) [62]. Occasionally, even a trilayer phase appears within the NaCl bilayer (Figure 3a and Figure 3b). Note also that dark protrusions originating from trapped Ar atoms are still visible through the NaCl island by STM as well as point defects. We do not exclude that these might be Cl vacancies [63] or CO molecules on NaCl, which will be investigated in future works. Figure 3d shows series of consecutive STM images of a NaCl island adsorbed on a terrace. Upon scanning with a tunneling resistance of about 10 GΩ, the entire island rotates under the tip action around a trilayer signature as pinning center. This is in contrast to those NaCl islands that are pinned to step edges. They remain always stable at *T* = 4.8 K, independent of the scanning conditions. As is, these islands exhibit characteristics similar to the ones on conventional metals [41,43–44]. Thus, they are likely adequate for the electronic decoupling of single atoms or molecules from the superconducting Pb(111).

### Single Fe atoms on Pb(111) and their lateral manipulations

**Figure 4:**
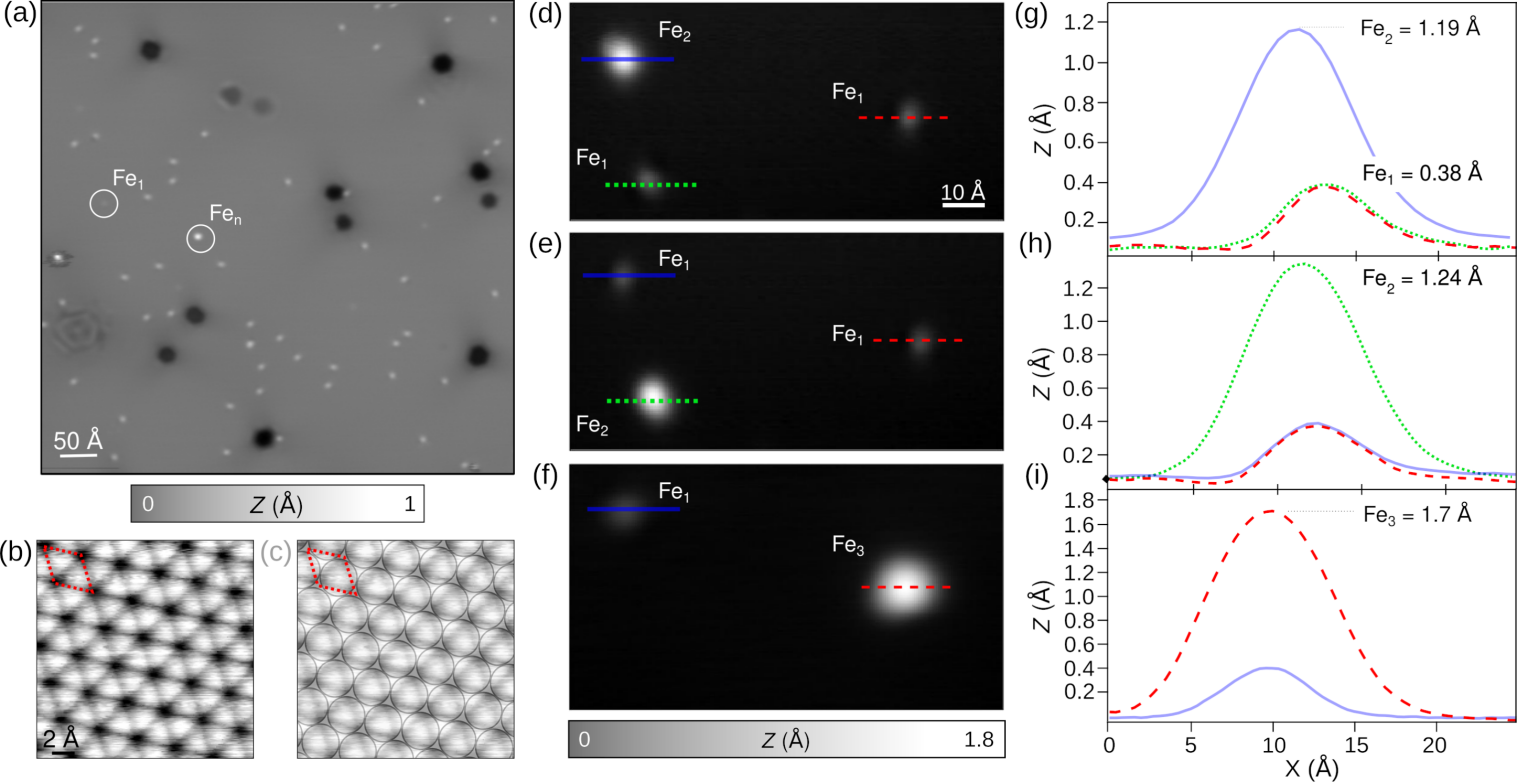
Fe adatoms on Pb(111) and their lateral manipulations. (a) STM overview image of Pb(111) after deposition of Fe adatoms (*V_t_* = −700 mV, *I_t_* = 5 pA). Fe_1_ and Fe*_n_* correspond to a single Fe adatom and clusters of *n* adatoms, respectively, (*V_t_* = −15 mV, *I_t_* = 5 pA). (b) Topographic STM image during the manipulation of a single Fe atom trapped in the STM junction. (c) Models of the Pb(111) corresponding to the STM image of panel (b). The red dashed parallelogram refers to the Pb(111) lattice. (d–f) Series of STM images of Fe adatoms and their successive lateral manipulations with the STM tip. In panel (f), the STM image shows the formation of a Fe trimer Fe_3_ by successive tip manipulations (imaging conditions, *V_t_* = −30 mV, *I_t_* = 60 pA). (g–i) Apparent STM heights extracted from panels (d–f) enabling one to distinguish from their topographic signatures Fe_1_, Fe_2_ and Fe_3_, respectively.

Figure 4 shows the deposition and controlled lateral manipulation of Fe adatoms on Pb(111). Upon deposition of Fe atoms on Pb(111) (kept below 15 K), several circular protrusions of different sizes and heights are observed by STM (Figure 4a). Their lateral sizes range from 0.3 to 1.5 Å, whereas their heights exhibit values of 0.4, 1.2 and 1.7 Å. Although no atomic resolution of these aggregates has been obtained, we interpret the variation of heights as a fingerprint for mostly Fe monomers, dimers and trimers, respectively (denoted as Fe_1_, Fe_2_ and Fe_3_, respectively, in the following). Note that we also do not exclude that few Fe adatoms are hydrogenated [64–65].

To confirm this assumption, we laterally manipulated single Fe adatoms with the STM tip [66–67] to intentionally form dimers and trimers and measure their apparent STM heights. To do so, the STM tip was positioned above a single Fe atom. The resistance of the STM junction was then decreased from about 50 GΩ (imaging) to 3 GΩ (manipulation) in order to trap the Fe atom in the STM junction [68]. Upon lateral tip displacements with a velocity of about 500 pm·s^−1^, the trapped Fe atom is successfully displaced over the surface. During this process, a so-called “atom manipulation image” [69] can be obtained from such dragging of the Fe atom over Pb(111) (Figure 4b). The geometric features resemble typical patterns observed in friction force microscopy (FFM) [28,38] or scanning tunneling hydrogen microscopy (SThM) [70–71], since the trapped Fe atom senses the surface potential in analogy to the probing tip of FFM. For clarity, we overlay the Pb(111) surface lattice on top of the image in Figure 4c. The darkest features are spaced by 0.35 Å in agreement with the lattice parameters of Pb(111) and likely correspond to hollow sites of the fcc structure of Pb(111). Indeed, we think that Fe atoms are preferentially adsorbed at these sites similar to Fe on Cu(111) [72].

Using this method, we transferred single atoms between different Fe clusters. In Figure 4d, two Fe single atoms (Fe_1_) and an assumed dimer (Fe_2_) are displayed. Figure 4g shows the corresponding apparent STM heights, which can be extracted from the solid, dotted and dashed lines of Figure 4d. Thus, we infer the heights of Fe_1_ and Fe_2_ aggregates to be *h*_1_ ≈ 0.4 Å and *h*_2_ ≈ 1.2 Å, respectively. As a verification, we then conducted the transfer of a single Fe atom from the Fe_2_ cluster to one surrounding Fe_1_ in order to form a new dimer. The result of such manipulation is shown in Figure 4e. Despite the exchange of Fe atoms by tip manipulation, the apparent height of Fe_1_ and Fe_2_ remains identical as demonstrated by the STM profile of Figure 4h.

Finally, we brought by two successive tip manipulations the atoms of Fe_2_ in Figure 4e to a third single atom. The resulting image (Figure 4f) reveals the formation of a Fe trimer (Fe_3_). Compared to the heights of Fe_1_ and Fe_2_, the height of Fe_3_ is about *h*_3_ = 1.7 Å. This evolution of STM apparent heights as a function of number of atoms in small Fe clusters is in good agreement with a similar study of Fe clusters on Cu(111) [72].

## Conclusion

Our results report on the systematic characterization by STM of the adsorption of carbon monoxide (CO), sodium chloride (NaCl) and iron adatoms (Fe) on the superconducting Pb(111) surface at low temperature (4.7 K). We show a surprising absence of STM topographic signatures of CO molecules on Pb(111), which we impute to their high propensity of diffusing under gentle scanning conditions. In contrast, CO molecules become apparent by STM on Pb(110), since they initiate attractive dipole–dipole interactions, which support the formation of linear aggregates. Furthermore, we show that deposition of NaCl on Pb(111) leads to bilayer islands similar to literature data. Lastly, cold-temperature deposition (≤15 K) of Fe on Pb(111) leads to the adsorption of adatoms and small Fe clusters. Using tip-induced lateral manipulations, we demonstrate the exchange of Fe single atoms between these clusters and characterize the variation of apparent STM height of each cluster as a function of the number of atoms. Overall, our findings provide new basic insights regarding the way to achieve high-resolution STM/AFM imaging with functionalized tips, decoupling of atoms or molecules and tip-induced lateral manipulation of Fe atoms above the prototypical Pb(111) superconducting surface.

## References

[1] Nayak C, Simon S H, Stern A, Freedman M, Das Sarma S (2008). Rev Mod Phys.

[2] Sato M, Ando Y (2017). Rep Prog Phys.

[3] Frolov S M, Manfra M J, Sau J D (2020). Nat Phys.

[4] Majorana E (1937). Nuovo Cim.

[5] Kitaev A Y (2001). Phys-Usp.

[6] Alicea J (2012). Rep Prog Phys.

[7] Wang Z, Rodriguez J O, Jiao L, Howard S, Graham M, Gu G D, Hughes T L, Morr D K, Madhavan V (2020). Science.

[8] Lutchyn R M, Bakkers E P A M, Kouwenhoven L P, Krogstrup P, Marcus C M, Oreg Y (2018). Nat Rev Mater.

[9] Mourik V, Zuo K, Frolov S M, Plissard S R, Bakkers E P A M, Kouwenhoven L P (2012). Science.

[10] Nadj-Perge S, Drozdov I K, Bernevig B A, Yazdani A (2013). Phys Rev B.

[11] Pientka F, Glazman L I, von Oppen F (2013). Phys Rev B.

[12] Klinovaja J, Stano P, Yazdani A, Loss D (2013). Phys Rev Lett.

[13] Nadj-Perge S, Drozdov I K, Li J, Chen H, Jeon S, Seo J, MacDonald A H, Bernevig B A, Yazdani A (2014). Science.

[14] Ruby M, Pientka F, Peng Y, von Oppen F, Heinrich B W, Franke K J (2015). Phys Rev Lett.

[15] Pawlak R, Kisiel M, Klinovaja J, Meier T, Kawai S, Glatzel T, Loss D, Meyer E (2016). npj Quantum Inf.

[16] Kim H, Palacio-Morales A, Posske T, Rózsa L, Palotás K, Szunyogh L, Thorwart M, Wiesendanger R (2018). Sci Adv.

[17] Jäck B, Xie Y, Li J, Jeon S, Bernevig B A, Yazdani A (2019). Science.

[18] Palacio-Morales A, Mascot E, Cocklin S, Kim H, Rachel S, Morr D K, Wiesendanger R (2019). Sci Adv.

[19] Fu L, Kane C L (2008). Phys Rev Lett.

[20] Sun H-H, Zhang K-W, Hu L-H, Li C, Wang G-Y, Ma H-Y, Xu Z-A, Gao C-L, Guan D-D, Li Y-Y (2016). Phys Rev Lett.

[21] Zhang P, Yaji K, Hashimoto T, Ota Y, Kondo T, Okazaki K, Wang Z, Wen J, Gu G D, Ding H (2018). Science.

[22] Zhu S, Kong L, Cao L, Chen H, Papaj M, Du S, Xing Y, Liu W, Wang D, Shen C (2020). Science.

[23] Kezilebieke S, Huda M N, Vaňo V, Aapro M, Ganguli S C, Silveira O J, Głodzik S, Foster A S, Ojanen T, Liljeroth P (2020). Nature.

[24] Eigler D M, Schweizer E K (1990). Nature.

[25] Stroscio J A, Eigler D M (1991). Science.

[26] Zeppenfeld P, Lutz C P, Eigler D M (1992). Ultramicroscopy.

[27] Langewisch G, Falter J, Fuchs H, Schirmeisen A (2013). Phys Rev Lett.

[28] Pawlak R, Kawai S, Meier T, Glatzel T, Baratoff A, Meyer E (2017). J Phys D: Appl Phys.

[29] Kawai S, Koch M, Gnecco E, Sadeghi A, Pawlak R, Glatzel T, Schwarz J, Goedecker S, Hecht S, Baratoff A (2014). Proc Natl Acad Sci U S A.

[30] Kawai S, Benassi A, Gnecco E, Söde H, Pawlak R, Feng X, Müllen K, Passerone D, Pignedoli C A, Ruffieux P (2016). Science.

[31] Stroscio J A, Celotta R J (2004). Science.

[32] Khajetoorians A A, Wegner D, Otte A F, Swart I (2019). Nat Rev Phys.

[33] Bartels L, Meyer G, Rieder K-H (1997). Appl Phys Lett.

[34] Gross L, Mohn F, Moll N, Liljeroth P, Meyer G (2009). Science.

[35] Gross L (2011). Nat Chem.

[36] Feldman B E, Randeria M T, Li J, Jeon S, Xie Y, Wang Z, Drozdov I K, Andrei Bernevig B, Yazdani A (2017). Nat Phys.

[37] Kamlapure A, Cornils L, Wiebe J, Wiesendanger R (2018). Nat Commun.

[38] Pawlak R, Ouyang W, Filippov A E, Kalikhman-Razvozov L, Kawai S, Glatzel T, Gnecco E, Baratoff A, Zheng Q, Hod O (2016). ACS Nano.

[39] Heinrich B W, Braun L, Pascual J I, Franke K J (2013). Nat Phys.

[40] Heinrich B W, Braun L, Pascual J I, Franke K J (2015). Nano Lett.

[41] Repp J, Meyer G, Stojković S M, Gourdon A, Joachim C (2005). Phys Rev Lett.

[42] Hirjibehedin C F, Lutz C P, Heinrich A J (2006). Science.

[43] Repp J, Steurer W, Scivetti I, Persson M, Gross L, Meyer G (2016). Phys Rev Lett.

[44] Meier T, Pawlak R, Kawai S, Geng Y, Liu X, Decurtins S, Hapala P, Baratoff A, Liu S-X, Jelínek P (2017). ACS Nano.

[45] Pawlak R, Vilhena J G, D’Astolfo P, Liu X, Prampolini G, Meier T, Glatzel T, Lemkul J A, Häner R, Decurtins S (2020). Nano Lett.

[46] Pawlak R, Drechsel C, D’Astolfo P, Kisiel M, Meyer E, Cerda J I (2020). Proc Natl Acad Sci U S A.

[47] Giessibl F J (2003). Rev Mod Phys.

[48] Nečas D, Klapetek P (2012). Cent Eur J Phys.

[49] Chang S H, Su W B, Jian W B, Chang C S, Chen L J, Tsong T T (2002). Phys Rev B.

[50] Jeffrey C A, Conrad E H, Feng R, Hupalo M, Kim C, Ryan P J, Miceli P F, Tringides M C (2006). Phys Rev Lett.

[51] Krupski A (2009). Phys Rev B.

[52] Schmid M, Hebenstreit W, Varga P, Crampin S (1996). Phys Rev Lett.

[53] Song S Y, Seo J (2017). Sci Rep.

[54] Auwärter W, Seufert K, Bischoff F, Ecija D, Vijayaraghavan S, Joshi S, Klappenberger F, Samudrala N, Barth J V (2012). Nat Nanotechnol.

[55] Choi D-J, Rubio-Verdú C, de Bruijckere J, Ugeda M M, Lorente N, Pascual J I (2017). Nat Commun.

[56] Fremy-Koch S, Sadeghi A, Pawlak R, Kawai S, Baratoff A, Goedecker S, Meyer E, Glatzel T (2019). Phys Rev B.

[57] Feng M, Cabrera-Sanfelix P, Lin C, Arnau A, Sánchez-Portal D, Zhao J, Echenique P M, Petek H (2011). ACS Nano.

[58] Briner B G, Doering M, Rust H-P, Bradshaw A M (1997). Science.

[59] Feng M, Lin C, Zhao J, Petek H (2012). Annu Rev Phys Chem.

[60] Ahner J, Mocuta D, Ramsier R D, Yates J T (1996). J Chem Phys.

[61] Kato H, Okuyama H, Ichihara S, Kawai M, Yoshinobu J (2000). J Chem Phys.

[62] Repp J, Meyer G, Rieder K-H (2004). Phys Rev Lett.

[63] Schuler B, Persson M, Paavilainen S, Pavliček N, Gross L, Meyer G, Repp J (2015). Phys Rev B.

[64] Mohr M, Weismann A, Li D, Brandbyge M, Berndt R (2021). Phys Rev B.

[65] Khajetoorians A A, Valentyuk M, Steinbrecher M, Schlenk T, Shick A, Kolorenc J, Lichtenstein A I, Wehling T O, Wiesendanger R, Wiebe J (2015). Nat Nanotechnol.

[66] Hla S-W, Braun K-F, Rieder K-H (2003). Phys Rev B.

[67] Hla S-W (2005). J Vac Sci Technol, B: Microelectron Nanometer Struct–Process, Meas, Phenom.

[68] Bartels L, Meyer G, Rieder K-H (1997). Phys Rev Lett.

[69] Celotta R J, Balakirsky S B, Fein A P, Hess F M, Rutter G M, Stroscio J A (2014). Rev Sci Instrum.

[70] Temirov R, Soubatch S, Neucheva O, Lassise A C, Tautz F S (2008). New J Phys.

[71] Weiss C, Wagner C, Kleimann C, Rohlfing M, Tautz F S, Temirov R (2010). Phys Rev Lett.

[72] Emmrich M, Huber F, Pielmeier F, Welker J, Hofmann T, Schneiderbauer M, Meuer D, Polesya S, Mankovsky S, Ködderitzsch D (2015). Science.

